# Progress Toward Regional Measles Elimination — Worldwide, 2000–2013

**Published:** 2014-11-14

**Authors:** Robert T. Perry, Marta Gacic-Dobo, Alya Dabbagh, Mick N. Mulders, Peter M. Strebel, Jean-Marie Okwo-Bele, Paul A. Rota, James L. Goodson

**Affiliations:** 1 Department of Immunization, Vaccines, and Biologicals, World Health Organization, Geneva, Switzerland; 2 Division of Viral Diseases, National Center for Immunization and Respiratory Diseases, CDC; 3 Global Immunization Division, Center for Global Health, CDC

In 2012, the World Health Assembly endorsed the Global Vaccine Action Plan* with the objective to eliminate measles in four World Health Organization (WHO) regions by 2015. Member states of all six WHO regions have adopted measles elimination goals. In 2010, the World Health Assembly established three milestones for 2015: 1) increase routine coverage with the first dose of measles-containing vaccine (MCV1) for children aged 1 year to ≥90% nationally and ≥80% in every district; 2) reduce global annual measles incidence to <5 cases per million; and 3) reduce global measles mortality by 95% from the 2000 estimate (1).† This report updates the 2000–2012 report (2) and describes progress toward global control and regional measles elimination during 2000–2013. During this period, annual reported measles incidence declined 72% worldwide, from 146 to 40 per million population, and annual estimated measles deaths declined 75%, from 544,200 to 145,700. Four of six WHO regions have established regional verification commissions (RVCs); in the European (EUR) and Western Pacific regions (WPR), 19 member states successfully documented the absence of endemic measles. Resuming progress toward 2015 milestones and elimination goals will require countries and their partners to raise the visibility of measles elimination, address barriers to measles vaccination, and make substantial and sustained additional investments in strengthening health systems.

## Immunization Activities

WHO and the United Nations Children’s Fund (UNICEF) use data from administrative records and surveys reported annually by member states to estimate coverage with MCV1 and the second dose of MCV (MCV2) through routine immunization services.§ Since 2003, member states also have reported the number of districts with ≥80% MCV1 coverage. Estimated MCV1 coverage increased globally from 73% to 83% from 2000 to 2009, then remained at 83%–84% through 2013 (Table 1). The number of member states with ≥90% MCV1 coverage increased from 84 (44%) in 2000 to 131 (68%) in 2012, then decreased to 129 (66%) in 2013. Among member states with ≥90% MCV1 coverage nationally, the proportion having ≥80% MCV1 coverage in all districts increased from 17% (18 of 104) in 2003 to 43% (56 of 131) in 2012, then declined to 37% (48 of 129) in 2013. Of the estimated 21.5 million infants not receiving MCV1 through routine immunization services in 2013, approximately 13.2 million (62%) were in six member states: India (6.4 million), Nigeria (2.7 million), Pakistan (1.7 million), Ethiopia (1.1 million), Indonesia (0.7 million), and the Democratic Republic of the Congo (0.7 million).

From 2000 to 2013, the number of member states providing MCV2 through routine immunization services increased from 96 (50%) to 148 (76%), with four member states introducing MCV2 in 2013. Estimated global MCV2 coverage increased from 15% in 2000 to 53% in 2013. During 2013, approximately 205 million children received MCV during supplementary immunization activities (SIAs) conducted in 34 member states.¶ Of these, 16 states (47%) reported ≥95% SIA coverage, and 21 (62%) provided one or more additional child health interventions during the SIA (Table 2).

## Disease Incidence

Countries report annually to WHO and UNICEF the number of measles cases from either case-based or aggregate surveillance systems.** Effective measles surveillance includes case-based surveillance with laboratory testing to confirm cases. In 2013, a total of 187 (96%)†† member states used case-based surveillance and 191 (98%)§§ had access to standardized quality-controlled testing through the WHO Measles and Rubella Laboratory Network.

During 2000–2013, the number of annual reported measles cases worldwide decreased 67%, from 853,479 to 279,776, and measles incidence decreased 72%, from 146 to 40 cases per million population (Table 1). The results for 2013 represent an increase from 227,739 reported cases and an incidence of 33 cases per million population in 2012, despite fewer member states reporting (189 in 2012 versus 176 in 2013).¶¶ The percentage of reporting member states with <5 cases per million increased from 64% in 2012 (120 of 189) to 66% in 2013 (116 of 176). During 2000–2013, the Region of the Americas maintained measles incidence at <5 cases per million.

The increase in measles incidence in 2013 largely was the result of outbreaks reported in the Democratic Republic of the Congo (89,108 cases), Nigeria (52,852), China (26,883), Pakistan (8,749), Angola (8,523), Indonesia (8,419), Uganda (7,878), Georgia (7,872), and Turkey (7,405). Reported cases in India declined from 33,634 in 2011 to 13,833 in 2013.

Genotypes of measles virus sequences were reported by 61 (56%) of the 108 member states reporting measles cases in 2013. Of 2,301 measles virus sequences reported to WHO,*** the genotype was B3 for 438 sequences (31 member states), D4 for 127 (19 member states), D8 for 1,555 (40 member states), D9 for 82 (13 member states), G3 for 15 (one member state) and H1 for 81 (nine member states). Five genotypes were reported in the Region of the Americas and WPR; three genotypes were reported in the Eastern Mediterranean Region (EMR), EUR and the South-East Asia Region; and one genotype was reported in the African Region (Table 1).

## Mortality Estimates

WHO has developed a model to estimate measles mortality in member states using numbers and age distribution of reported cases, routine and SIA MCV coverage, and age-specific, country-specific case-fatality ratios (3,4). New measles vaccination coverage and case data for all member states during 2000–2013 led to a new series of mortality estimates. During this period, estimated measles deaths decreased 75%, from 544,200 to 145,700, and all regions had substantial reductions in estimated measles mortality (Table 1). Compared with no measles vaccination, an estimated 15.6 million deaths were prevented by measles vaccination during 2000–2013 (Figure).

## Regional Verification of Measles Elimination

By 2013, RVCs had been established in the Region of the Americas, EUR, EMR, and WPR, and all RVCs have met except for EMR. The annual RVC report from the Region of the Americas indicated the region continues to have multiple measles virus importations, whereas three member states in WPR and 16 member states in EUR have documented the absence of endemic measles virus transmission to their RVC.

### Discussion

During 2000–2013, coverage worldwide with both routine doses of MCV combined with SIAs contributed to a 72% decrease in reported measles incidence and a 75% reduction in estimated measles mortality. The decrease in measles mortality was one of three main contributors (along with decreases in pneumonia and diarrhea) to the decline in overall mortality in children aged <5 years and to progress toward the fourth Millennium Development Goal††† (5). During this period, measles vaccination prevented an estimated 15.6 million deaths. RVCs in EUR and WPR verified that 19 member states have successfully documented the absence of endemic measles. However, based on current trends of measles vaccination coverage and incidence, the WHO Strategic Advisory Group of Experts on Immunization concluded that the 2015 global targets will not be achieved on time; little progress has been made toward measles elimination in EMR and EUR, and progress in WPR is at risk (6).

The Democratic Republic of the Congo, Ethiopia, India, Indonesia, Nigeria, and Pakistan, together accounted for 28% of global population but >60% of children not reached with MCV1, and >70% of estimated global measles deaths in 2013. In these member states, child health systems will need to be strengthened to ensure that their immunization programs reach ≥95% of children with 2 MCV doses through routine immunization services and high-quality SIAs.

The findings in this report are subject to at least three limitations. First, MCV coverage estimates are affected by inaccurate estimates of the size of target populations, inaccurate reporting of doses delivered, and reporting of SIA doses given to children outside the target group. Second, underestimation in surveillance data can occur because not all patients with measles seek care and not all of those who seek care are reported. Finally, some member states report aggregate, unconfirmed cases rather than case-based data.

To achieve measles elimination, all the strategies described in the Global Vaccine Action Plan and the 2012–2020 Global Measles and Rubella Strategic Plan (8) of the Measles & Rubella Initiative will need to be implemented.§§§ Policy and practice gaps leading to missed opportunities for measles vaccination will need to be addressed, such as the reluctance of vaccinators to open 10-dose vials when few children are present or to vaccinate children aged ≥12 months through routine immunization services, and inappropriate contraindications to vaccination. The verification process (9) to document the absence of endemic measles virus in member states can be implemented in the African Region, South-East Asia Region, and EMR, and used to raise awareness of and advocate for solutions to programmatic gaps. To resume progress toward achieving the 2015 Millennium Development Goals, global measles control targets, and regional measles elimination goals, the visibility of measles elimination activities needs to be increased and investments made to strengthen health systems and achieve equitable access to immunization services.

**What is already known on this topic?**

During 2000–2009, global vaccination coverage with the first dose of measles-containing vaccine (MCV1) increased from 72% to 83%, and annual measles incidence decreased from 146 reported cases per million population in 2000 to 41 cases per million in 2009. During 2009–2012, MCV1 coverage remained at 83%–84%, the number of member states providing a second dose of measles-containing vaccine (MCV2) through routine immunization services increased from 134 (69%) to 144 (74%), and approximately 693 million children were vaccinated against measles during SIAs. Measles elimination in four of six WHO regions by 2015 is among the objectives of the Global Vaccine Action Plan.

**What is added by this report?**

During 2000–2013, an estimated 15.6 million deaths were prevented by measles vaccination. The number of member states providing MCV2 through routine immunization services increased to 148 (76%) in 2013, and global MCV2 coverage was 53%. During 2013, a total of 205 million children were vaccinated against measles during supplementary immunization activities. Large outbreaks continued in the Democratic Republic of the Congo (89,108 cases), India (13,822 cases), and Pakistan (8,749 cases), and new outbreaks were reported from Nigeria (52,852), and China (26,883).

**What are the implications for public health practice?**

The African, Eastern Mediterranean, and European regions are not progressing as expected to achieve their elimination targets, and the Western Pacific Region is at risk. To accelerate progress toward achieving these regional measles elimination targets, policy and practice gaps preventing reaching greater numbers of children will need to be addressed, visibility of measles elimination efforts increased, and adequate resources provided to strengthen health systems and achieve the objectives of the Global Vaccine Action Plan.

## Figures and Tables

**FIGURE:**
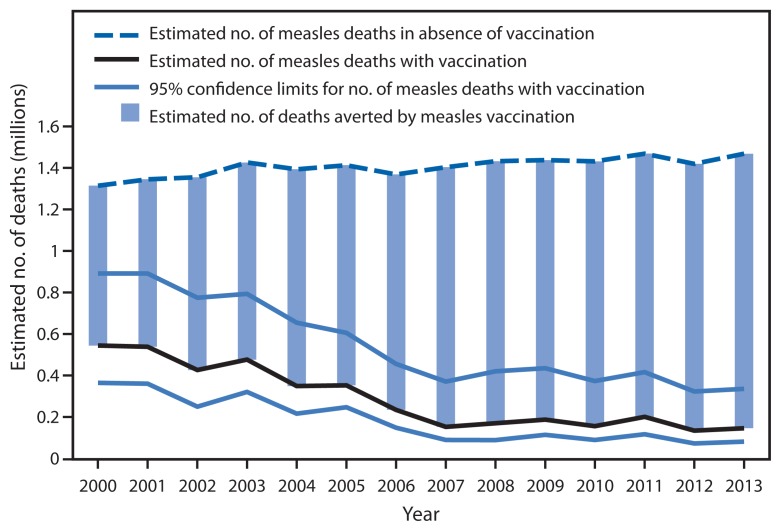
Estimated number of measles deaths and number of deaths averted by measles vaccination — worldwide, 2000–2013

**TABLE 1:** Estimates of coverage with the first dose (MCV1) and second dose (MCV2) of measles-containing vaccine administered through routine immunization services among children aged 1 year, reported measles cases and incidence, by World Health Organization (WHO) region, 2000 and 2013

WHO region	2000							

	% coverage with MCV1*	% member states with coverage ≥90%	% coverage with MCV2	No. of reported measles cases†	Measles incidence (cases per million population)§¶	% member states with incidence <5 per million	Estimated no. of measles deaths	

							No.	(95% CI)
African	53	9	5	520,102	841	8	342,300	(224,600–570,600)
Americas	93	63	45	1,754	2.1	89	<100	—
Eastern Mediterranean	72	57	28	38,592	90	17	54,100	(32,900–87,600)
European	91	60	48	37,421	50	48	300	(100–1,500)
South-East Asia	65	30	3	78,558	51	0	137,100	(101,000–184,100)
*South-East Asia (excluding India)*	78	—	9	39,723	80	0	52,300	*(32,700*–*80,300)*
India	59	—	0	38,835	37	0	84,700	(68,200–103,700)
Western Pacific	85	43	2	177,052	105	30	10,400	(5,800–47,700)
**Total**	**73**	**43**	**15**	**853,479**	**146**	**38**	**544,200**	**(364,300–891,500)**

WHO region	2013												

	% coverage with MCV1*	% member states with coverage ≥90%	% coverage with MCV2	No.of reported measles cases†	% decline from 2000	Measles incidence (cases per million population)§¶	% decline from 2000	% member states with incidence <5 per million	Reported measles geno-types**	Estimated measles deaths		% mortality reduction 2000 to 2013	% total measles deaths in 2013

										No.	(95% CI)		
African	74	32	7	171,905	67	185	78	53	B3	74,200	(41,600–165,000)	84	51
Americas	92	80	46	490	72	0.5	76	100	B3, D4 D8, D9, H1	<100		—	0
Eastern Mediterranean	78	57	65	20,885	46	35	61	32	B3, D4, D8	32,500	(18,600–61,900)	49	22
European	95	87	82	24,689	34	32	37	71	B3, D4, D8	100	(0–1,200)	65	0
South–East Asia	78	55	53	30,101	62	16	68	45	D4, D8, D9	37,500	(20,800–67,100)	63	26
*South–East Asia (excluding India)*	89	60	78	16,279	59	27	66	50		10,000	(3,300–27,300)	74	7
India	74	–	42	13,822	64	11	70	0		27,500	(17,500–39,800)	57	19
Western Pacific	97	81	92	31,706	82	17	84	68	B3, D8, D9, G3, H1	1,500	(100–40,100)	88	1
**Total**	**84**	**66**	**53**	**279,776**	**67**	**40**	**72**	**66**		**145,700**	**(81,100–335,400)**	**75**	**100**

**Abbreviations:** CI = confidence interval; UNICEF = United Nations Children’s Fund.

* Based on WHO/UNICEF estimates of national immunization coverage, available at http://apps.who.int/immunization_monitoring/globalsummary/timeseries/tswucoveragemcv.html.

† Based on WHO reported measles case data, available at http://apps.who.int/immunization_monitoring/globalsummary/timeseries/tsincidencemeasles.html. Data for Region of the Americas available at http://www.paho.org/hq/index.php?option=com_docman&task=doc_view&itemid=270&gid=27446&lang=en.

§ Based on World Population Prospects: the 2013 Revision (CD-Rom edition). New York, United Nations Organization, Population Division, Department of Economic and Social Affairs, 2013.

¶ Any country not reporting data on measles cases for that year was removed from both the numerator and denominator.

** Reported to the Measles Nucleotide Surveillance (MeaNS) database, available at http://www.who-measles.org.

**TABLE 2:** Measles supplementary immunization activities (SIA) and delivery of other child health interventions, by World Health Organization (WHO) region and member state, 2013

			Children reached in targeted age group		
				
WHO region/Member state	Age group targeted	Extent of SIA*	No.	(%)†	Other interventions delivered
**Africa**					
Botswana	9–59 mos	National	198,341	(94)	
Cape Verde	9 mos–24 yrs	National	240,166	(95)	rubella vaccine
Central African Republic	9–59 mos	National	691,233	(87)	oral polio vaccine, vitamin A, anthelmintics
Comoros	9–59 mos	National	86,516	(86)	vitamin A, anthelmintics, TT vaccine
Republic of the Congo	6–59 mos	National	726,979	(92)	anthelmintics
DRC	9 mos–9 yrs 9 mos–14 yrs	Rollover (national)§	12,160,677	(101)	oral polio vaccine, vitamin A, anthelmintics
Ethiopia	9–59 mos	National	11,609,484	(98)	oral polio vaccine
Ghana	9 mos–14 yrs	National	11,062,605	(99)	rubella vaccine
Lesotho	9–59 mos	National	147,676	(72)	oral polio vaccine, vitamin A, anthelmintics
Madagascar	9–59 mos	National	3,316,542	(92)	anthelmintics, TT vaccine
Malawi	9–59 mos	National	2,405,018	(105)	oral polio vaccine, vitamin A, anthelmintics
Mozambique	6–59 mos	National	4,078,637	(102)	anthelmintics
Nigeria	6–59 mos 9–59 mos	National	31,777,071	(94)	oral polio vaccine, anthelmintics
Rwanda	9 mos–14 yrs	National	4,391,081	(103)	rubella and oral polio vaccines, vitamin A, anthelmintics
Senegal	9 mos–14 yrs	National	6,097,123	(101)	rubella vaccine
South Africa	6–59 mos	National	4,186,192	(100)	oral polio vaccine
Swaziland	6–59 mos	National	119,207	(97)	oral polio vaccine, vitamin A, anthelmintics
Togo	9 mos–9 yrs	Rollover (national)§	1,641,635	(96)	vitamin A, anthelmintics
**Americas**					
Guatemala	1–5 years	National	1,659,469	(91)	mumps, rubella and oral polio vaccines, vitamin A, anthelmintics
**Eastern Mediterranean**					
Afghanistan	9–59 mos	Subnational	875,874	(85)	oral polio and TT vaccines
Iraq	6–12 yrs	National	5,563,532	(96)	
Jordan	9 mos–14 yrs				
6 mos–19 yrs	National	4,000,936	(102)	rubella and oral polio vaccines, vitamin A	
Lebanon	9 mos–18 yrs				
9 mos–14 yrs	National	662,616	(88)	rubella vaccine	
Morocco	9 mos–19 yrs	National	10,191,571	(91)	rubella vaccine
Pakistan	9 m–9 yrs	Sindh and Punjab	30,988,259	(97)	oral polio vaccine
Somalia	9–59 mos	Subnational child health days and SIAs in newly accessible areas	744,077	(85)	oral polio vaccine, vitamin A, anthelmintics, TT vaccine
Sudan	9 mos–14 yrs	National	14,976,050	(98)	oral polio vaccine, vitamin A, anthelmintics
Syria	6–10 yrs				
12–15 yrs	Subnational	1,549,105	(80)	rubella and mumps vaccines	
Yemen	6 mos–10 yrs	Subnational	283,687	(93)	
**European**					
Georgia	2–14 yrs	National	31,385	(49)	rubella and mumps vaccines
**South-East Asia**					
India	9 months–10 years	Rollover (national)§	33,640,721	(82)	
**Western Pacific**					
Cambodia	9 mos–14 yrs	National	4,576,633	(105)	vitamin A, anthelmintics, rubella vaccine
Micronesia	12–47 mos	National	3,435	(95)	rubella and mumps vaccines
Vanuatu	12–59 mos	National	33,604	(102)	rubella vaccine
**Total**			**204,718,027**		

**Abbreviations:** TT = tetanus toxoid; DRC = Democratic Republic of the Congo.

* SIAs generally are carried out using two approaches. An initial, nationwide catch-up SIA targets all children aged 9 months to 14 years; it has the goal of eliminating susceptibility to measles in the general population. Periodic follow-up SIAs then target all children born since the last SIA. Follow-up SIAs are generally conducted nationwide every 2–4 years and generally target children aged 9–59 months; their goal is to eliminate any measles susceptibility that has developed in recent birth cohorts and to protect children who did not respond to the first measles vaccination. The exact age range for follow-up SIAs depends on the age-specific incidence of measles, coverage with 1 dose of measles-containing vaccine, and the time since the last SIA.

† Values >100% indicate that the intervention reached more persons than the estimated target population.

§ Rollover national campaigns started the previous year or will continue into the next year.

## Footnotes

* The Global Vaccine Action Plan is the implementation plan of the Decade of Vaccines, a collaboration between WHO; UNICEF; the Bill and Melinda Gates Foundation; Gavi, the Vaccine Alliance; the U.S. National Institute of Allergy and Infectious Diseases; the African Leaders Malaria Alliance; and others to extend, by 2020 and beyond, the full benefit of immunization to all persons. Additional information is available at http://www.who.int/immunization/global_vaccine_action_plan/en and at http://apps.who.int/gb/ebwha/pdf_files/wha65/a65_22-en.pdf.

† Whereas the coverage milestone is to be met by every member state, the incidence and mortality reduction milestones are to be met globally.

§ For MCV1, among children aged 1 year or, if MCV1 is given at age ≥1 year, among children aged 24 months. For MCV2, among children at the recommended age of administration of MCV2, as per the national immunization schedule. WHO/UNICEF estimates of national immunization coverage are available at http://www.who.int/immunization_monitoring/routine/immunization_coverage/en/index4.htm.

¶ SIAs generally are carried out using two target age ranges. An initial, nationwide catch-up SIA targets all children aged 9 months–14 years, with the goal of eliminating susceptibility to measles in the general population. Periodic follow-up SIAs then target all children born since the last SIA. Follow-up SIAs generally are conducted nationwide every 2–4 years and target children aged 9–59 months; their goal is to eliminate any measles susceptibility that has developed in recent birth cohorts and to protect children who did not respond to the first measles vaccination.

** Available at http://apps.who.int/immunization_monitoring/globalsummary/timeseries/tsincidencemeasles.html.

†† Member states without case-based measles surveillance in 2013 were Djibouti, India, Mauritius, Seychelles, Sao Tome and Principe, Somalia, and South Sudan.

§§ Member states without access to standardized quality-controlled testing by the WHO Measles and Rubella Laboratory Network in 2013 included Cape Verde, Sao Tome and Principe, and Seychelles.

¶¶ Countries not reporting in 2012 were Kenya from the African Region and Finland, France, Malta, and Uzbekistan from EUR. In 2013, countries not reporting were Libya and the United Arab Emirates from EMR, Austria, Bosnia and Herzegovina, Ireland, Italy, Malta, Monaco, San Marino, and Ukraine from EUR and Brunei Darussalam, Cook Islands, Fiji, the Marshall Islands, Nauru, Samoa, Singapore, and Tuvalu from WPR.

*** Sequences were for the 450 nucleotide carboxy-terminal of the nucleocapsid gene in the measles virus genome. Genotypes isolated from three cases of subacute sclerosing panencephalitis (D3, D6, and D7) were excluded from the total. Data as of October 7, 2014 from the Measles Nucleotide Surveillance (MeaNS) database, available at http://www.who-measles.org.

††† Additional information available at http://www.un.org/millenniumgoals.

§§§ The Measles & Rubella Initiative is a partnership established in 2001 as the Measles Initiative, led by the American Red Cross, CDC, the United Nations Foundation, UNICEF, and WHO. Additional information is available at http://www.measlesrubellainitiative.org.
